# The future of telerehabilitation: embracing virtual reality and augmented reality innovations

**DOI:** 10.11604/pamj.2024.47.157.42956

**Published:** 2024-04-03

**Authors:** Waqar Mohsin Naqvi, Ifat Waqar Naqvi, Gaurav Vedprakash Mishra, Vishnu Diwakar Vardhan

**Affiliations:** 1 Department of Physiotherapy, College of Health Sciences, Gulf Medical University, Ajman, United Arab Emirates,; 2 Faculty of Health Professions Education, Datta Meghe Institute of Higher Education and Research, Wardha, India,; 3 Ravi Nair Physiotherapy College, Datta Meghe Institute of Higher Education and Research, Wardha, India

**Keywords:** Tele-rehabilitation, virtual reality, augmented reality

## Abstract

The integration of virtual reality (VR) and augmented reality (AR) into the telerehabilitation initiates a major change in the healthcare practice particularly in neurological and also orthopedic rehabilitation. This essay reflects the potential of the VR and AR in their capacity to create immersive, interactive environments that facilitate the recovery. The recent developments have illustrated the ability to enhance the patient engagement and outcomes, especially in tackling the complex motor and cognitive rehabilitation needs. The combination of artificial intelligence (AI) with VR and AR will bring the rehabilitation to the next level by enabling adaptive and responsive treatment programs provided through real-time feedback and predictive analytics. Nevertheless, the issues such as availability, cost, and digital gap among many others present huge obstacles to the mass adoption. This essay provides a very thorough review of the existing level of virtual reality and augmented reality in rehabilitation and examines the many potential gains, drawbacks, and future directions from a different perspective.

## Essay

Virtual reality (VR) and augmented reality (AR) have given birth to a new era of telerehabilitation, a giant leap toward health innovation. A celebrated feature of VR and AR is their ability to institute many engaging and dynamic experiences, which contributes to the redefinition of many therapeutic approaches, especially within neurology and orthopedics [1]. They go beyond traditional therapeutic techniques by providing patients with immersive environments, which greatly improves treatment efficiency. The purpose of this essay is to further examine the considerable contribution and contemporary status of VR and AR in rehabilitation, a field covering a wide scope of applications and struggling with the issues it is currently facing within the healthcare environment. Unlike other technologies, the integration of VR and AR into rehabilitation has many challenges. Barriers such as high setup costs, ongoing maintenance demands, and the digital divide, especially in under-resourced areas, impede widespread uptake. Furthermore, ethical issues related to data privacy and security have arisen in this data-centric method [2,3]. This discussion provides a balanced view of potential advantages and obstacles of VR and AR in rehabilitation, emphasizing the importance of patient-focused, ethical, and practical applications of these technologies [4]. The deployment of VR and AR in telerehabilitation introduces innovative opportunities for patient care and enhances accessibility, customization, and immersion in treatment methodologies. Emphasizing ethical development and application is crucial to ensure that these technologies positively impact patient outcomes and quality of life [5].

**Recent advances in virtual reality and augmented reality:** significant advancements in VR and AR technologies have broadened the scope of rehabilitation treatments for neurological and orthopedic conditions. Virtual reality technology has undergone rapid development, leading to greater realism and immersion. Its application in rehabilitation has evolved into complex, interactive environments that closely mimic real-life scenarios, proving especially beneficial in stroke rehabilitation by assisting patients in regaining motor skills through virtual exercises [6]. Similarly, AR has been pivotal in augmenting motor skills and cognitive functions by overlaying digital information onto the physical world to create engaging experiences. Augmented reality applications offer immediate feedback and coaching for individuals with Parkinson's, thereby enhancing motor function and coordination [7]. The integration of AI with VR and AR represents a significant leap forward, customizing rehabilitation plans based on patient progress and ensuring treatments are tailored to individual needs, thereby increasing the efficiency and effectiveness of rehabilitation [8]. Such tools are unavoidable in motivating patients because they can transform monotonous exercises into fun activities. The involvement of rehabilitation protocols in the adherence and outcome of recovery has been unequivocally demonstrated.

However, a few challenges are yet to be taken, the key one being making VR and AR tech on demand as well as affordability measures. Currently, the focus targets involve offering equipment-related solutions to these problems, considering the cost efficiency and user-friendliness of the VR/AR gear. In addition, more attention has been paid to the prevention of harmful effects, such as motion sickness, acquired from VR, to benefit all users. The future of VR and AR in rehabilitation looks very promising and will probably happen from the attempts to make the simulation more realistic, to the development of the systems in which the wearable devices are more integrated into the body, and finally to the personalization of the treatment with the use of improved and AI algorithms [9].

**Personal insights and opinions:** the incorporation of virtual reality (VR) and augmented reality (AR) in telerehabilitation led to the advancement of healthcare technology with the introduction of both new opportunities and challenges. The highly immersive and interactive characteristics of virtual reality (VR) and augmented reality (AR) offer many possibilities for rehabilitation, especially for conditions that require repetitive and monotonous treatment regimens. These technologies have the potential to revolutionize the patient experience, resulting in a much more engaging rehabilitation, wherein motivation and adherence might also be enhanced. However, the problem lies in ensuring equal distribution and usage effectiveness because many challenges have to be addressed, such as accessibility, cost, and the need for healthcare professional training. Future VR and AR rehabilitation will be huge as long as continuous improvement in cost-effectiveness and accessibility is made, and ethical issues are handled [10].

**Potential benefits and challenges:** the integration of VR and AR into telerehabilitation offers numerous potential benefits but also presents significant challenges that require careful consideration for effective implementation. Benefits include making rehabilitation programs more engaging, providing realistic environments for better rehabilitation outcomes, allowing personalized therapy, and facilitating data collection for therapy optimization [11,12]. However, challenges, such as ensuring accessibility and affordability, managing technical complexity, maintaining patient safety and comfort, and addressing ethical concerns, remain critical barriers to their widespread adoption [13,14].

**The future of virtual reality and augmented reality in rehabilitation:** with further development of VR and AR technologies, they will guide many changes in rehabilitation across several sectors. Further advancement is expected to lead to increasingly complex, user-oriented systems, facilitate access and delivery of highly individual rehabilitation programs, step up telerehabilitation services, and increase cross-disciplinary cooperation. Furthermore, ethical guidelines and regulatory development will play a vital role in guaranteeing that these technologies will be used responsibly to protect patient privacy and data security [15]. Such a visionary approach reveals the potency of virtual and augmented reality to revolutionize rehabilitation, thereby emphasizing the need for innovation, partnership, and ethics to unlock their complete potential.
